# In Vitro Assessment of the Cytotoxic and Antiproliferative Profile of Natural Preparations Containing Bergamot, Orange and Clove Essential Oils

**DOI:** 10.3390/molecules27030990

**Published:** 2022-02-01

**Authors:** Vlad Tiberiu Alexa, Atena Galuscan, Codruța M. Soica, Antoanela Cozma, Dorina Coricovac, Florin Borcan, Iuliana Popescu, Alexandra Mioc, Camelia Szuhanek, Cristina Adriana Dehelean, Daniela Jumanca

**Affiliations:** 1 Department of Preventive, Community Dentistry and Oral Health, Faculty of Dental Medicine, “Victor Babeş” University of Medicine and Pharmacy, Eftimie Murgu Sq. No. 2, 300041 Timisoara, Romania; vlad.alexa@umft.ro (V.T.A.); galuscan.atena@umft.ro (A.G.); jumanca.daniela@umft.ro (D.J.); 2 Orthodontic Research Center (ORTHO-CENTER), Faculty of Dental Medicine, “Victor Babeş” University of Medicine and Pharmacy, Eftimie Murgu Sq. No 2, 300041 Timisoara, Romania; szuhanek.camelia@umft.ro; 3 Translational and Experimental Clinical Research Center in Oral Health (TEXC-OH), Department of Preventive, Community Dentistry and Oral Health, “Victor Babeş” University of Medicine and Pharmacy 14A TudorVladimirescu Ave., 300173 Timisoara, Romania; 4 Department of Pharmaceutical Chemistry, Faculty of Pharmacy, “Victor Babeş” University of Medicine and Pharmacy, Eftimie Murgu Sq. No. 2, 300041 Timisoara, Romania; alexandra.petrus@umft.ro; 5 Research Center for Pharmaco-Toxicological Evaluations, Faculty of Pharmacy “Victor Babeș” University of Medicine and Pharmacy Timisoara, Eftimie Murgu Square No. 2, 300041 Timișoara, Romania; dorinacoricovac@umft.ro (D.C.); cadehelean@umft.ro (C.A.D.); 6 Department of Soil Science, Faculty of Agriculture, Banat’s University of Agricultural Sciences and Veterinary Medicine “King Michael I of Romania” from Timisoara, Calea Aradului No. 119, 300641 Timisoara, Romania; iuliana_popescu@usab-tm.ro; 7 Department of Toxicology, Faculty of Pharmacy, “Victor Babeş” University of Medicine and Pharmacy, Eftimie Murgu Sq. No. 2, 300041 Timisoara, Romania; 8 Department of Analytical Chemistry, Faculty of Pharmacy, “Victor Babeş” University of Medicine and Pharmacy, Eftimie Murgu Sq. No. 2, 300041 Timisoara, Romania; fborcan@umft.ro; 9 Department of Orthodontics, Faculty of Dental Medicine, “Victor Babeş” University of Medicine and Pharmacy, Eftimie Murgu Sq. No 2, 300041 Timisoara, Romania

**Keywords:** cell viability, *Citrus bergamia*, *Citrus sinensis*, emulsion, immortalized human keratinocytes, human melanoma cells (A375), human primary gingival fibroblasts (HGF), human squamous cell carcinoma (SCC-4), *Syzygium aromaticum*

## Abstract

Medicinal plants and essential oils (EOs), in particular, were intensively studied in recent years as viable alternatives for antiproliferative chemical synthetic agents. In the same lines, the present study focuses on investigating the effects of natural preparations (emulsions) based on EOs obtained from *Citrus bergamia* Risso (bergamot-BEO), *Citrus sinensis* Osbeck (orange-OEO), and *Syzygium aromaticum* Merill et L. M. Perry (clove-CEO) on different healthy (human immortalized keratinocytes—HaCaT and primary human gingival fibroblasts—HGF) and human tumor cell lines (human melanoma—A375 and oral squamous carcinoma—SCC-4) in terms of the cells’ viability and cellular morphology. The obtained results indicate that the CEO emulsion (ECEO) induced a dose-dependent cytotoxic in both healthy (HaCaT and HGF) and tumor (A375 and SCC-4) cells. OEO emulsion (EOEO) increased cell viability percentage both for HaCaT and A375 cells and had an antiproliferative effect at the highest concentration in HGF and SCC-4 cells. BEO emulsion (EBEO) decreased the viability percentage of SCC-4 tumor cells. By associating OEO with CEO as a binary mixture in an emulsified formulation, the inhibition of tumor cell viability increases. The E(BEO/OEO) binary emulsion induced an antiproliferative effect on oral health and tumor cells, with a minimal effect on skin cells. The non-invasive tests performed to verify the safety of the test compound’s emulsions at skin level indicated that these compounds do not significantly modify the physiological skin parameters and can be considered safe for human skin.

## 1. Introduction

According to the GLOBOCAN 2020 report estimates, emitted by the International Agency for Research on Cancer (IARC), in 2020, 19.3 million new cases of cancer as well as approximatively ten million cancer-related deaths were reported. In the light of these data, it could be stated that cancer is rated as a leading cause of death worldwide [1]. Cancer is defined as an intricate multistep disease characterized by genomic instability (changes in key genes and epigenetic alterations) that leads to uncontrolled cell growth, resistance to cell death, invasion, metastasis, and angiogenesis. Although the chemotherapeutic arsenal was significantly improved in recent years by the discovery of novel agents such as protein kinases inhibitors and monoclonal antibodies, there are several major drawbacks, such as: lack of specificity, severe adverse effects, and resistance to therapy, can diminish their efficacy [2]. Therefore, there is a current need for more effective and biocompatible therapeutics and/or preventive anticancer alternatives that can be implemented in clinical practice.

Increased interest was attributed in the last years to natural compounds with antitumoral potential. Plant-derived essential oils are one of the most studied alternative approaches for cancer prophylaxis/treatment [3].

Essential oils (EOs) are hydrophobic liquid mixtures usually having a lower density of water and comprising versatile natural compounds that are separated using different approaches [4]. Traditional and advanced methods for the extraction of the EOs were used from the past to the present. The traditional method of extracting EOs is hydrodistillation (HD) or steam distillation (SD), followed by gas chromatography-mass spectroscopy (GC-MS) identification and characterization of chemical compounds [5,6]. The disadvantages of the classical extraction methods are given by the higher amounts of samples necessary for extraction, thermal changes during the extraction process, the possibility of compounds oxidation due to the longer extraction times [7]. Compared with the HD extraction, SD shows some superiority, including shorter extraction times, lower levels of oxidation and chemical changes of natural compounds, less energy use, and a lower probability of losing more polar compounds. However, as with HD, SD extraction yields are often lower [8]. Today, modern EOs extraction techniques are used, such as: microwave-assisted techniques such as microwave-assisted hydrodistillation (MAHD), microwave steam distillation (MSD), solvent-free microwave extraction (SFME). These techniques are more times saving and efficient compared to the classical hydrodistillation approach [8,9].

Traditionally, EOs are used for their biological activities, including antiseptic, analgesic, sedative, anti-inflammatory, spasmolytic, and anesthetic properties [3]. In the last decade, in order to test their possible use as an alternative or complementary anticancer treatment, several EOs have been investigated in terms of cytotoxic and antiproliferative effects, both in vitro, on cancer cell lines as well as in vivo by using tumor-bearing experimental animals as an alternative or complementary anticancer treatment [3]. Different mechanisms have been suggested for the reported cytotoxic effects of EOs or their components, including induction of cell death through apoptosis and/or necrosis, cell cycle arrest, and loss of key organ function [3]. Some of these effects are attributable to the lipophilic nature and the low molecular weights of the constituent elements of EOs that allow them to cross cell membranes by altering their phospholipid layers, increasing membrane fluidity, and leading to the leakage of ion and cytoplasmic content. Reduced ATP production, alteration of pH gradient, and loss of mitochondrial potential are just a few of the consequences of cellular membrane disturbances. Furthermore, essential oils can also act as pro—or antioxidants, thus affecting the cellular redox state [3].

Bergamot (*Citrus bergamia* Risso) essential oil (BEO) is widely used in the cosmetic industry [10], as well as in the biomedical field due to its antimicrobial [11], antifungal [12], and anti-helminthic potential, as well as the phytocomplex capacity to increase oxidative metabolism in human polymorphonuclear leukocytes [13]. The antiproliferative potential of BEO has been tested on cancer cell lines such as human neuroblastoma [14] and colorectal cancer [15] and also on healthy dermal fibroblasts [16] in order to assess its biocompatibility.

Oranges (*Citrus sinensis* Osbeck) are among the most commonly produced fruit crops in the world. The orange essential oil (OEO) is extracted from the peels of the fruit, which are discarded in the process of orange juice production. Previous studies have demonstrated the antiproliferative in vitro effect of EOs extracted from various types of oranges on different types of cancer cells. Thus, OEO extracted from Gannanzao orange peel has been shown to inhibit the proliferation of liver cancer (HepG2), colon cancer (HCT116) [17], lung cancer (A549), and prostate cancer cells (22RV1) [18].

In the current context of promoting the ‘circular economy’ based on waste recovery, the use of orange or bergamot peels to obtain EOs containing active principles with therapeutic purposes represents a scientific and applied approach of high impact at a European level.

Clove (*Syzygium aromaticum* Merill et L.M. Perry) essential oil (CEO) is widely used, especially as topical applications for a wide variety of medical purposes. Its main component, eugenol, is known to have antibacterial, antifungal, anti-inflammatory [19], and antioxidant properties [20]. In terms of anti-inflammatory activity, studies on human cell lines have shown a potentially beneficial effect of CEO in treating periodontal disease due to its inhibitory effect on interleukin 6 in gingival fibroblasts [21]. Koh et al. (2013) have shown the anti-inflammatory activity of eugenol in human gingival fibroblast and dental pulp cells [22]. Han and Parker (2017) provided important evidence of the anti-inflammatory and tissue remodeling activity induced by CEO in human dermal fibroblasts [23]. Controversially, Prashar et al. (2006) reported that CEO exhibited significant cytotoxicity against human fibroblasts and endothelial cells at concentrations as low as 0.03% (*v*/*v*) up to 73% of this effect attributable to its major component, eugenol [24]; the authors recommended it is used with caution, in diluted forms, in particular for topical application.

Arung ET et al. (2011) demonstrated that eugenol and its acetate derivative inhibit melanin formation in B16 melanoma cells [25]. A study by Liu H. et al. (2014) showed the antiproliferative effect of clove on several human cancer cell lines, including: breast (MCF-7), ovarian (SKOV-3), cervical (HeLa), liver (BEL-7402), pancreatic (PANC-1), and colon (HT-29) cells [26]. Stepping on previous results regarding the cytotoxic potential of EOs on different tumor cell lines, the present study aims to study the impact of bergamot, orange, and clove essential oils formulated as emulsions and their blends: (i) in vitro by assessing the potential cytotoxicity on healthy and tumor cell lines of different origin, as immortalized human keratinocytes (HaCaT), human melanoma cells (A375), human primary gingival fibroblasts (HGF), and human squamous cell carcinoma (SCC-4), and (ii) at skin level by evaluating the physiological skin parameters, as transepidermal water loss (TEWL), erythema and hydration using non-invasive methods.

The originality of this study is, on the one hand, the testing of mentioned EO_S_ mixtures on some not yet exploited cell lines, on the other hand, to evaluate the possibility of using, in the context of the circular economy, some wastes (orange and bergamot peels) as active principles from EO_S_ in medical applications.

## 2. Results and Discussion

### 2.1. GC-MS Composition of EO_S_

The chemical composition of the analyzed binary mixtures of EOs is presented in Table 1. The results showed that BEO/OEO contains D-limonene as the major component (43.32%), followed by α-pinene (26.50%), while CEO/OEO has D-limonene (29.82%) and p-eugenol is the predominant compound (32.71%). The ternary mixture highlighted the individual compositions of the binary mixture. In BEO/CEO/OEO, 16 compounds were identified, of which D-limonene represents 32.69%, α-pinene 20.15%, and eugenol 15.85% of the total compositions. The ternary mixture ensures a balanced composition in active principles that can act in the sense of increasing the antiproliferative effects. The chemical composition of EOs has been shown to be essential in determining their antiproliferative character. The study of the CEO, OEO, and BEO composition has been previously reported by our group [27].

Multiple studies focused on the chemical compounds found in the EOs composition of OEO, BEO, and CEO [28,29,30,31,32,33,34]. According to these studies, the main chemical compound of OEO was D-limonene in different percentages depending on the extraction method, genetic differences, environmental factors, and harvesting practices. The concentration of D-limonene varies between 77.49% [30], 68–98% [29] and 1–14% [28]. Other compounds such as linalool acetate, linalool, farnesol, and β-pinene were also reported in OEO [27]. In BEO, D-limonene (60.44%) and γ-terpinene (20.28%) were the major compounds identified [33], but also linalool (33.9–77.1%) and linalyl acetate (2.2–45.4%) were identified [34].

### 2.2. The Impact of EOs Emulsions on the Viability of Healthy Human Cells Andimmortalized Keratinocytes Morphology—HaCaT

**Figure 1:**
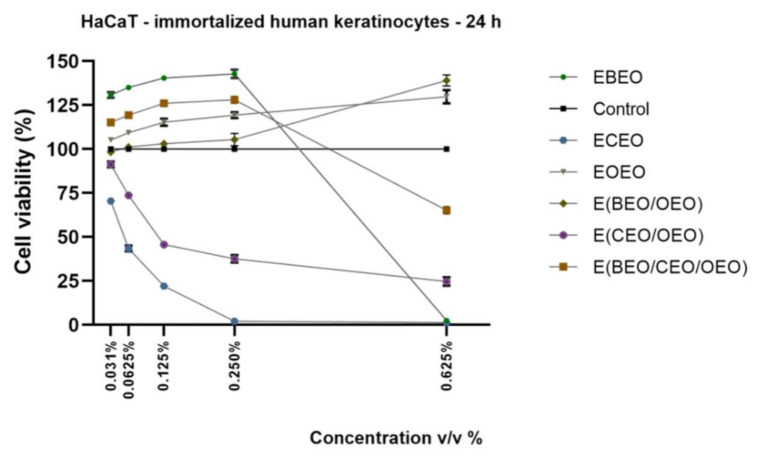
In vitro viability assessment of Eos emulsions (ECEO, EBEO, EOEO) and their mixtures (E(BEO/OEO), E(CEO/OEO) and E(BEO/CEO/OEO)) in HaCaT—at 24 h post-stimulation by Alamar blue assay. The results are expressed as cell viability percentage (%) normalized to control (unstimulated cells). The data represent the mean values ± SD of three independent experiments.

HaCaT is a nontumorigenic immortalized monoclonal cell line that resists long-term culture growth without supplementary growth factors. These cells present normal morphogenesis and exert functional activities as isolated keratinocytes. HaCaT cell line is considered to be a reliable model for the assessment of different skin disorders, such as: skin inflammation, angiogenesis [35], and skin carcinogenesis [36,37], and is frequently used in the literature to assess the biocompatibility of different EOs [38,39]. In the present study, HaCaT cells were exposed for 24 h to different concentrations (0.031%, 0.0625%, 0.125%, 0.250% and 0.625% *v*/*v*) of EOs emulsions (EBEO, ECEO, and EOEO) and their combinations E(BEO/OEO), E(CEO/OEO) and E(BEO/CEO/OEO) and evaluated in terms of cell viability percentage and morphological changes. Our results indicated that EBEO stimulation had no toxic impact on HaCaT cells’ viability at low concentrations (≤0.250% *v*/*v*), but at the highest concentration tested (0.625% *v*/*v*) induced a strong cytotoxic effect, the percentage of viable cells reaching 2.43% (Figure 1). ECEO proved a dose-dependent cytotoxic effect on HaCaT cells viability, the calculated percentage of viable cells at the highest concentrations being lower than 5% (2.07% for 0.250% and 1.317% for 0.625%, respectively) (Figure 1). All the concentrations tested for EOEO did not trigger a reduction of cells viability. Moreover, a stimulatory effect was observed with increasing concentration. A similar effect was observed in the case of binary emulsion E(BEO/OEO). The binary emulsion E(CEO/OEO) determined a concentration-dependent reduction of HaCaT cells viability percentage, the lowest percentages calculated were at the highest concentrations (0.250–37.61% and 0.625–24.64%, respectively). The ternary emulsion E(BEO/CEO/OEO) induced a stimulatory effect at low concentrations (≤0.250%), but the highest concentration used a significantly reduced cells’ viability percentage (65.23%) (Figure 1).

**Figure 2:**
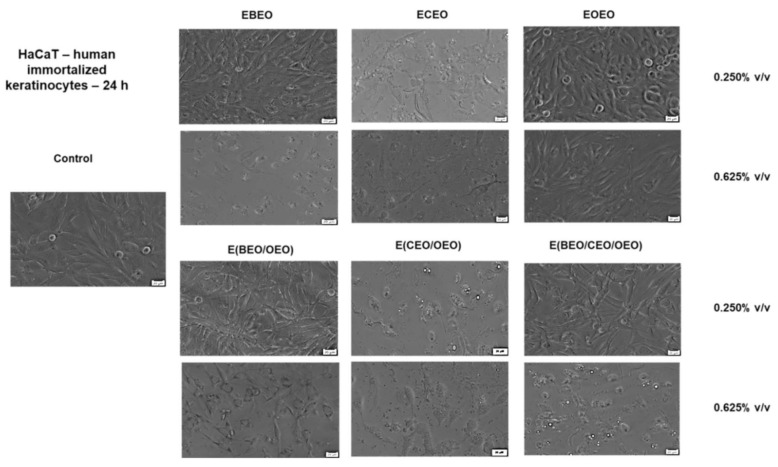
The morphological aspect of HaCaT—in culture: Control—unstimulated cells and cells stimulated with different test compounds: ECEO, EOEO, EBEO, E(BEO/OEO), E(CEO/OEO), and E(BEO/CEO/OEO) for 24 h. The scale bar is 20 µM.

The cell viability results were in line with the changes recorded in cells morphology after 24 h stimulation with the tested products (Figure 2). The control cells (unstimulated HaCaT) exhibited a fibroblastic-like shape, high confluence, and adherence to the culture plate. ECEO stimulation triggered significant changes in cell morphology, such as damaged cells with shrunken aspects, round cells floating in the medium, and the presence of cell debris. Similar changes were noticed in the case of binary emulsion E(CEO/OEO) but at a lower extent as compared to ECEO. Modification of shape, presence of round cells floating, and a low confluence were also noticed in the case of EBEO and E(BEO/CEO/OEO) stimulation, but only at the highest concentration tested (0.625% *v*/*v*); these experimental data that can be correlated with the low percentage of viable cells were recorded for the respective samples. The other tested compounds, EOEO, E(OEO/BEO), EBEO (0.250% *v*/*v*), and E(BEO/CEO/OEO) (0.250% *v*/*v*) did not interfere with HaCaT cell morphology; moreover, the tested cells showed similar characteristics with the control cells (Figure 2).

### 2.3. The Impact of the First Compounds on Human Primary Gingival Fibroblasts HGF Viability and Morphology

The primary human gingival fibroblasts—HGF cells used in the present study were obtained (according to manufacturer’s description—ATCC—American Type Culture Collection) from a Caucasian female (60 years) gingival tissue and present the following characteristics: a spindle shape-type morphology that adheres to the culture plate and is also bipolar and retractile.

**Figure 3:**
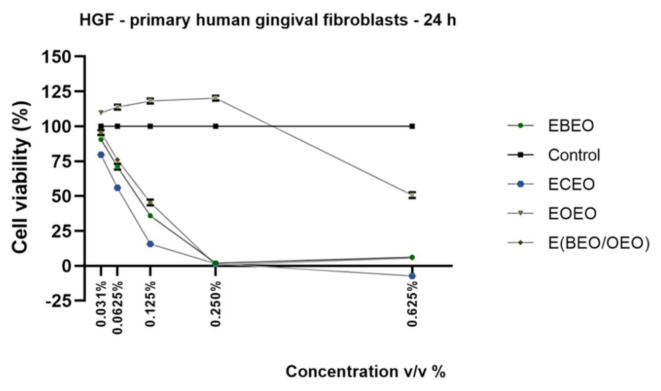
In vitro viability assessment EOs emulsions (EBEO, ECEO, and EOEO) and their mixture E(BEO/OEO) in HGFat 24 h post-stimulation by Alamar blue assay. The results are expressed as cell viability percentage (%) normalized to control (unstimulated cells). The data represent the mean values ± SD of three independent experiments.

Since the test preparations (ECEO, EOEO, EBEO and their binary and ternary emulsion mixtures) are intended for oral use, their impact on healthy cells originating from the oral cavity was assessed; the human primary gingival fibroblast cell line was selected for this evaluation. To assess the impact of test compounds on HGF cells’ viability and morphology was applied under the same experimental protocol as described for HaCaT cells. After 24 h stimulation, the following data were recorded: (i) ECEO, EBEO, and E(BEO/OEO) showed a concentration-dependent reduction of cells viability (viability percentages <5% at the highest concentrations tested), still the most cytotoxic was ECEO (0.031% *v*/*v*–79.60% to 0.625–7.22% viable cells) (Figure 3), (ii) EOEO reduced HGF cells’ viability only at the highest concentration (0.625% *v*/*v*–50.68% viable cells) and (iii) the impact of binary E(CEO/OEO) and ternary E(BEO/CEO/OEO) preparations was also assessed, but after 24 h stimulation, gelatinous sediment was formed in the respective wells, which interfered with the viability assay.

**Figure 4:**
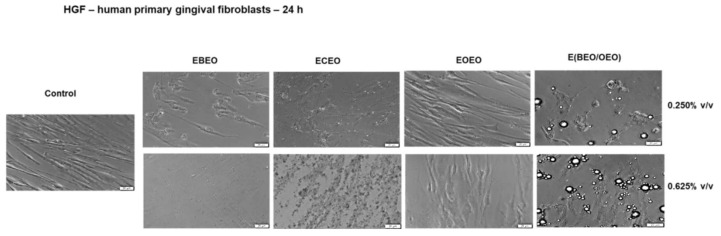
The morphological aspect of HGF in culture: Control unstimulated cells and cells stimulated with different test compounds: ECEO, EBEO, EOEO, and EBEO/OEO) for 24 h. The scale bar is 20 µM.

The addition of test compounds (EBEO, ECEO, and E(BEO/OEO)) determined significant changes in HGF cells morphology such as cell debris, shrunken and floating cells, signs specifically for cytotoxicity, and cell death. HGF cells stimulated with EOEO exhibited similar morphological traits (spindle-shaped and adherent) as those described for control (unstimulated) cells, with merely slight shape changes after the addition of 0.625% *v*/*v* test sample (Figure 4). These findings support the data recorded in the viability assay.

### 2.4. The Impact of the First Compounds on Human Melanoma Cells—A375 Viability and Morphology

A375 human melanoma cell line is one of the most frequently used melanoma cell lines for research studies [37], recording over 2300 citations in the PubMed database at present. This cell line was derived from a primary skin melanoma with an epithelioid morphology and expresses two mutant genes, namely BRAF (one of the most prevalent mutations in melanoma tumors) and CDKN2, genes that are associated with melanoma of sun-damaged skin. In addition, A375 cells preserve the features of the human genitor typical for cutaneous melanoma, which makes them valid for in vivo models [40].

**Figure 5:**
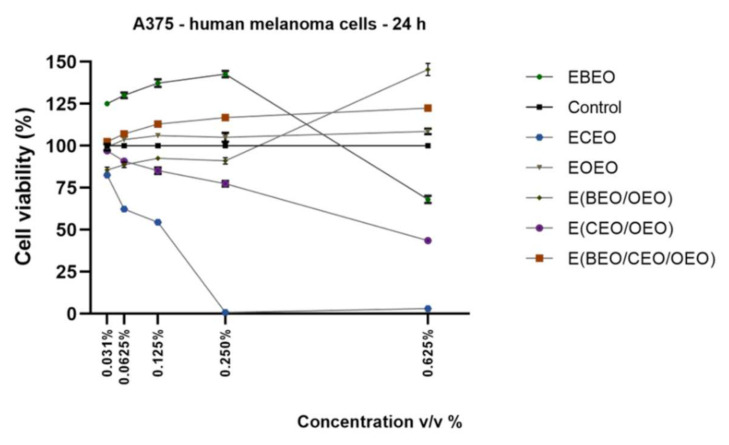
In vitro viability assessment of EOs emulsions (EBEO, ECEO, and EOEO) and their mixtures (E(BEO/OEO), E(CEO/OEO), and E(BEO/CEO/OEO)) onA375—cells at 24 h post-stimulation by Alamar blue assay. The results are expressed as cell viability percentage (%) normalized to control (unstimulated cells).

Stimulation of A375 cells with test samples led to the following results: (i) EBEO displayed a stimulatory effect at low concentrations (≤0.250% *v*/*v*) whereas the highest concentration 0.625% *v*/*v* reduced significantly melanoma cells’ viability (68.11%), (ii) ECEO induced a dose-dependent cytotoxic effect the viability percentages calculated for the highest concentrations used being <5% (0.250% *v*/*v*–0.83% and 0.625% *v*/*v*–3.01% viable cells, respectively), (iii) EOEO, E(BEO/OEO) and E(BEO/CEO/OEO) induced a stimulatory effect with increasing concentration and (iv) E(CEO/OEO) showed dose-dependent cytotoxicity but at a lower extent as ECEO (0.250% *v*/*v*–77.47% and 0.625% *v*/*v*–43.58% viable cells) (Figure 5).

**Figure 6:**
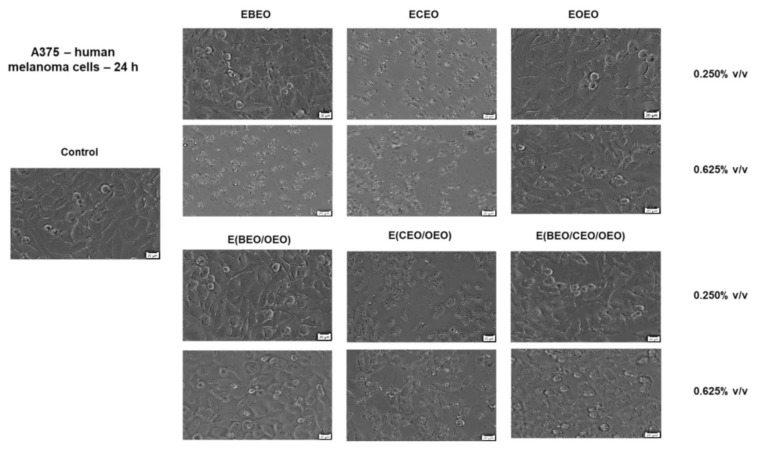
The morphological aspect of A375 cells in culture: Control—unstimulated cells and cells stimulated with different test compounds: ECEO, EOEO, EBEO, E(BEO/OEO), E(CEO/OEO), and E(BEO/CEO/OEO) for 24 h. The scale bar is 20 µM.

As described for the healthy cell lines analyzed, the impact of the first compounds on A375 cells’ morphological features was also assessed. The most significant changes in cells’ morphology were observed at the highest concentrations tested (Figure 6) and support the observations provided by the cell viability assay. A decreased confluence, the presence of detached and round cells, and cell debris were noticed after EBEO (0.625% *v*/*v*), ECEO (0.250% and 0.625% *v*/*v*) and E(CEO/OEO) (0.250% and 0.625% *v*/*v*) stimulation. The other tested samples—EBEO (0.250% *v*/*v*), EOEO, E(BEO/OEO), and E(BEO/CEO/OEO) did not affect A375 cells’ morphology, their aspect being similar to the control cells (unstimulated cells) (Figure 6).

### 2.5. The Impact of the First Compounds on Human Squamous Tongue Carcinoma Cells—SCC-4 Viability and Morphology

**Figure 7:**
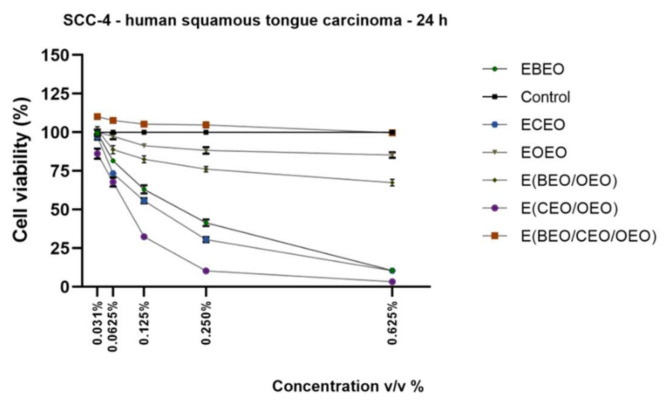
In vitro viability assessment of EOs emulsions (EBEO, ECEO, and EOEO) and their mixtures E(BEO/OEO), E(CEO/OEO), and E(BEO/CEO/OEO)) in SCC-4 cells at 24 h post-stimulation by Alamar blue assay. The results are expressed as cell viability percentage (%) normalized to control (unstimulated cells). The data represent the mean values ± SD of three independent experiments.

A common pathology of the oral cavity is represented by tongue carcinoma; therefore, one of the objectives of this study was to determine the potential antiproliferative effects of the test compounds and their mixture against this type of cells. SCC-4 are cancerous cells with an epithelial-like morphology isolated from a male diagnosed with tongue squamous cell carcinoma (description offered by the manufacturer—ATCC). Our viability results indicate that all test compounds and their binary mixtures exerted a concentration-dependent decrease in SCC-4 viability percentages, as follows: E(CEO/OEO) (3.26% viable cells at 0.625% *v*/*v*) > ECEO (10.49% viable cells) > EBEO (10.53% viable cells) > E(BEO/OEO) (67.46% viable cells) > EOEO (85.35%). The ternary emulsion induced a stimulatory effect at low doses (0.031% *v*/*v*–110.21 % viable cells), which started to decrease with increasing concentration (0.625% *v*/*v*–99.69% viable cells) (Figure 7).

**Figure 8:**
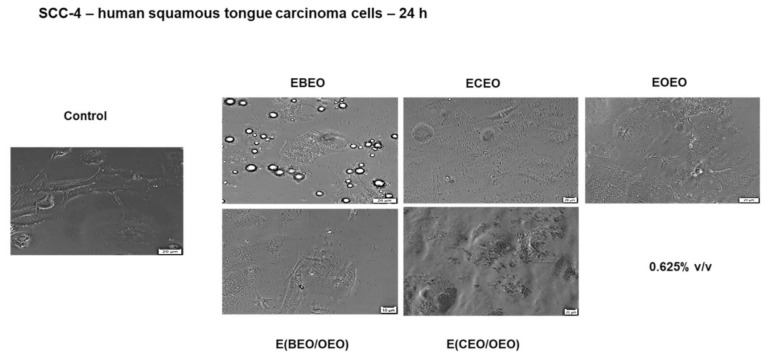
The morphological aspect of SCC-4 in culture: Control unstimulated cells and cells stimulated with different test compounds: ECEO, EOEO, EBEO, E(BEO/OEO), E(CEO/OEO) for 24 h. The scale bar is 20 µM.

The viability results are supported by the cell morphology findings that indicate significant changes in cells shape such as lower confluence, as well as cell debris and floating cells after the application of ECEO, E(BEO/OEO), and E(CEO/OEO); similar traits can be noticed after the OEO stimulation, but in a lower extent (Figure 8).

Essential oils such as bergamot (BEO), clove (CEO), and orange (OEO) present multiple beneficial effects (antimicrobial, antifungal, antiproliferative, etc.) being widely applied in cosmetics, pharmaceutics, and food industry [41,42], still in recent years several concerns were raised regarding the safety of these oils [24,43]. Nevertheless, a comprehensive review published in 2018 emphasized the negligible toxicity of all Citrus essential oils; in addition, the authors reached the conclusion that Citrus essential oils can be safely used in the industry of food and beverages, medicine, and cosmetics [44]. Only phototoxicity was shown as adverse effect after the topical application of certain essential oils, such as bergamot [45]. However, this phototoxic effect could be used as a treatment alternative against lentigo maligna or lentigo malignant melanoma [45].

To correct these inconveniences, several new formulations were proposed to increase the efficacy of these EOs and to minimize their toxicity [46,47].

On the same lines, the present study proposed the assessment of lecithin-based emulsions of BEO, CEO, and OEO essential oils and their binary and ternary mixtures in different healthy and tumor cell lines. The EBEO treatment determined different responses within the cells, as follows: (a) a stimulatory effect at low doses (≤0.250% *v*/*v*) and a cytotoxic one at the highest dose (0.625% *v*/*v*) in HaCaT and A375 cells (Figure 1 and Figure 5) and (b) a dose-dependent cytotoxic effect in HGF and SCC-4 cells (Figure 3 and Figure 7). Previous studies have emphasized the cytotoxic activity of BEO, which can be enhanced by nanoemulsification, in particular, if polysorbates are used as emulsifiers [16]; therefore, BEO emulsion could be used as an anticarcinogenic agent but cannot act as a food preservative. BEO (0.01%) was implied as strongly anti-inflammatory, antiproliferative, and cytotoxic in human dermal fibroblasts by inhibiting proteins related to inflammation, immune reactions, and tissue-remodeling processes [4]. Different authors have suggested that the necrotic and apoptotic cell death induced by BEO are related to the activation of multiple pathways by its phytocomponents; however, individual phytocomponents such as limonene were not able to inhibit cell viability, thus suggesting an enhanced cytotoxic activity between phytocompounds found in BEO [48]. Indeed, synergistic-stimulated autophagy was identified against SH-SY5Y neuroblastoma cells, attributed to the two major components in bergamot oil, D-limonene and linalyl acetate [49,50]. Menichini et al. [45] reported that BEO induced a photo-cytotoxic effect in A375 cells, data that supports our results.

EOEO treatment induced a stimulatory effect in HaCaT and A375 cells (Figure 1 and Figure 5), whereas in the case of SCC-4 cells, a concentration-dependent decrease (Figure 7) was recorded. The results presented in Figure 3 show that, regarding the effect of EOs emulsion on HGF cells, only the highest concentration was cytotoxic. The low skin toxicity of orange essential oil that has as main component D-limonene is supported by other results from the literature, such as the report of Erhan M.K. (2020), which showed a lack of a cytotoxic effect of D-limonene in HaCaT cells after 24 h exposure to 0.08% D-limonene [51].

Contrary to our results, one previously published study revealed a very strong cytotoxic activity of orange essential oil on HaCaT cells which recommends it as a natural anti-inflammatory and anticancer agent [52]; however, previous studies of the same author indicated a change of activity following the entry in metabolic processes. OEO exerted cytotoxic effects on two cancer cell lines, such as MCF7 (breast cancer), HCT116 (colorectal carcinoma), and one non-carcinogenic cell line (HSF, human foreskin fibroblasts) [18] in a dose-dependent manner; the authors suggested its use in anticancer treatments. Similar results were obtained by Yang et al. (2017), who reported the antiproliferative activity of orange oil containing limonene against A549 human lung cancer cells and 22RV-1 prostate cancer cells [18]. An antiproliferative effect of the orange essential oil against A375 cells was also reported [46,53] but at higher concentrations than the ones tested in the present study.

In the present study, the ECEO emulsion proved to be the most cytotoxic compound independent of the cell type tested (Figure 1, Figure 3, Figure 5 and Figure 7). Previous data reported the toxicity of clove oil at a concentration of 0.03% on human skin cells, toxicity attributed to eugenol [22]. Clove oil proved to be cytotoxic for mouse fibroblast cell line (3T3) [41], non-cancer human fibroblasts (MRC-5) (IC50 = 15.75 ± 2.4 μg/mL) [54], HEL 12469 human embryo lung cells [55] and HaCaT cells [39], data that are in agreement with our results. Clove oil and its main component, eugenol, have already been investigated as anticarcinogenic agents in prostate and oral squamous cancers [56]. Han et al. (2017) reported the antiproliferative activity of clove oil on human dermal fibroblasts through the inhibition of several pro-inflammatory markers; in addition, clove oil interfered with the regulation of signaling pathways with a pivotal role in inflammation and cancer development [23]. Another in vitro study revealed the cytotoxic properties of clove oil and of its main component, eugenol, as well against human fibroblasts and endothelial cells, even when used in very small concentrations (0.03%); the authors established that eugenol was responsible for 73% of clove oil’s cytotoxic effect, thus suggesting the presence of additional cytotoxic compounds in its composition [24]. The data regarding the effect of clove essential oil in melanoma are rather scarce. Still, eugenol showed strong antimelanoma properties [57,58,59,60]. As regards the impact of CEO in human squamous cancer cells, several studies showed that eugenol presented a cytotoxic effect on oral squamous carcinoma cells at a similar dose that triggered cytotoxicity in healthy cells (primary gingival fibroblast—HGF) [61] and modified the metabolic profile of oral squamous cancer cells [62].

The binary emulsion E(BEO/OEO) had a stimulatory effect in HaCaT cells (Figure 1) and a dose-dependent cytotoxic effect in HGF and SCC-4 cells (Figure 3 and Figure 7). An interesting finding was observed in the case of A375 cells (Figure 5) at low concentrations 0.031% *v*/*v* the viability percentage was reduced to 85.58%, whereas by increasing the concentration, the percentage of viable cells also increased. This behavior could be explained as a hormetic effect, but further investigations are needed in order to confirm this hypothesis.

The E(CEO/OEO) binary emulsion determined a dose-dependent decrease in all cells’ viability (HaCaT and SCC-4 cells), but at a lower extent in the case of A375 cells (Figure 1, Figure 5 and Figure 7).

The coefficient of drug interaction (CDI) values was calculated for each binary and ternary association of natural preparations in order to evaluate the properties of two/three EO combinations; the results are described in Table 2 for each type of cell.

The results show that, when applied on HaCaT cells, the binary associations increase the inhibition of cell proliferation. Thus, suggesting the presence of synergism between the associated EO, while the ternary association reduces the inhibitory effect on cell proliferation. Consequently, binary combinations should be used with caution in topical applications, given the cytotoxic effects on healthy dermal cells. Similar conclusions can be drawn regarding oral fibroblasts when the BEO/OEO combination is applied. When the A375 melanoma cell line is tested, the combination E(BEO/OEO) in the lower concentrations increases the antiproliferative effect while all other associations in either concentration reduce the antiproliferative effect of a single EO; therefore, E(BEO/OEO) is recommended as anticancer agent in melanoma treatment but without the advantage of selectivity, due to the simultaneous cytotoxic effect on healthy dermal cells. On SCC4 tongue carcinoma cells, the synergic antiproliferative effect occurs only for the E(CEO/OEO) and E(BEO/OEO) combinations, thus recommending it as an anticancer agent; however, the same synergic activity on HaCaT reduces its selectivity against cancer cells. The ternary BEO/CEO/OEO association induces additive or antagonistic effects on both healthy and cancer cell lines; therefore, it cannot act as an anticancer agent but can be safely applied on dermal or oral tissue, thus being recommended as a component of foods and beverages.

### 2.6. Skin Evaluation

**Figure 9:**
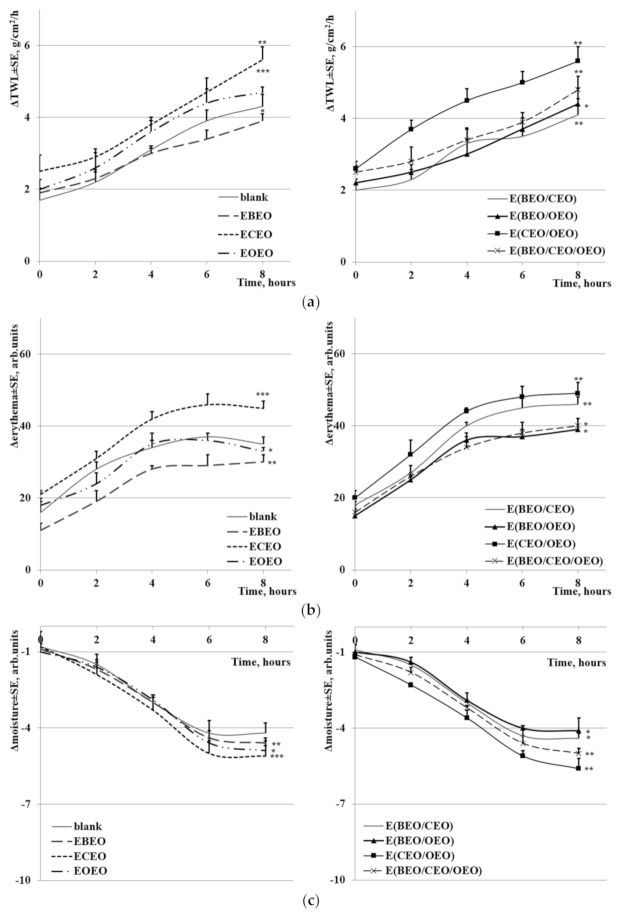
Irritant test: (**a**) loss of transdermal water (TWL), (**b**) erythema, (**c**) level of hydration of the stratum corneum (* *p* ≤ 0.05, ** *p* ≤ 0.01, *** *p* ≤ 0.001).

In order to evaluate the safety of skin application of EOs natural preparations, several specific tests were conducted: (a) loss of transdermal water (TWL), (b) erythema, and (c) level of hydration of the stratum corneum (Figure 9).

Compared to a blank test sample without EOs, after 8 h of topical contact, EOEO and EBEO exhibit lower and ECEO higher TWL values; all samples of binary and ternary mixtures provided a higher loss of transdermal water than the control sample (Figure 9a).

The erythema values can be correlated with the content of hemoglobin. When mono-component emulsions were tested, the highest erythema values were recorded for ECEO (higher than control), followed by EOEO and EBEO (lower than control). The erythema values for binary and ternary emulsions after 8 h of application varied as follows: E(CEO/OEO) > E(BEO/CEO) > E(CEO/OEO/BEO) > E(BEO/OEO) (Figure 9b).

Figure 9c displays the hydration of the stratum corneum after sample application. A significant decrease in hydration, lower than control, was noticed after the application of mono-component emulsions for 8 h: E(CEO > OEO > BEO). The binary and ternary emulsions showed a similar profile, with a minimum of hydration recorded for E(CEO/OEO), followed by E(BEO/CEO/OEO), E(BEO/CEO), and E(BEO/OEO), respectively.

The assessment of the irritating potential through relatively fast test procedures (5 evaluations/8 h) showed that in all skin tests, the level of TWL and erythema increases while the hydration of the stratum corneum decreases. However, these changes are minimal, thus indicating the test samples are safe for human skin treatments.

Previous studies highlighted that the physiological skin parameters (TEWL, erythema, and skin hydration) did not show an irritant or toxic effect following application of EOs [63,64].

## 3. Materials and Methods

### 3.1. GC-MS Characterization of EOs

Commercial essential oils (CEO, OEO, and BEO) were purchased from Solaris (SC Solaris Plant SRL, Bucharest, Romania (44°42′38″ N 25°99′86″ E). The binary mixtures BEO/OEO and CEO/OEO were prepared in 1:1 (*v*/*v*) ratio, respectively ternary mixture BEO/CEO/OEO in 1:1:1 (*v*/*v*/*v*) ratio.

The GS/MS QP 2010 Plus (Shimadzu, Kyoto, Japan) and the capillary column AT WAX 30 m × 0.32 mm × 1μm (Santa Clara, CA, USA) were used for chemical characterization of EOs. The column temperature was kept at 40 °C for 1 min, programmed to 210 °C at a rate of 5 °C/min., and kept constant at 210 °C for 5 min. The carrier gas was Helium at a rate of 1 mL/min. The injection was performed at a split ratio of 1:50, and injection volume was 1 μL. Injector and ion source temperatures were 250 °C and 220 °C, respectively. To identify the compounds, the NIST 02, Wiley 275 libraries spectra library has been used. The linear retention indices (LRI) were determined in relation to a homologous series of *n*-alkanes (C8–C24) under the same operating conditions. All injections were performed in triplicate. The analysis was carried out within Interdisciplinary Research Platform of BUAS.

### 3.2. Preparation Procedure for Natural Emulsions Based on Essential Oils

The natural emulsions based on EOs analyzed in the present study were obtained as direct emulsions oil-in-water (O/W) using lecithin as an emulsifying agent. The methodology, the composition, and the physicochemical properties of natural preparation were reported in our previous study [65].

In brief, the emulsions were prepared in water, and the EO was mixed using an Ultrasonic Processor VCX130 PB 130 Watt, Frequency 20 kHz, (Sonics & Materials INC., Newtown, CT, USA), for 10 min at an amplitude of 98%. All samples contained 6 mg lecithin (Walmart, Rogers, AR, USA). Six natural preparations (5% stock concentration) were obtained, as follows: (1) E(CEO) (emulsion containing CEO); (2) E(OEO) (emulsion containing OEO); (3) E(BEO) (emulsion containing BEO); (4) E(BEO/OEO) (emulsion containing BEO and OEO); (5) E(CEO/OEO) (emulsion containing CEO and OEO); and (6) E(BEO/CEO/OEO) (emulsion containing BEO, CEO, and OEO).

### 3.3. Cell Lines

The cell lines used in the present study were: HGF—human primary gingival fibroblasts (ATCC^®^ PCS-201-018™), SCC-4—human squamous cell carcinoma cell line (ATCC^®^ CRL-1624™), HaCaT—immortalized human keratinocytes (CLS Cell Lines Service GmbH), and A375—human melanoma cells (ATCC^®^ CRL-1619™).

For cell culture and cell viability assay, the following reagents were needed: specific culture medium—Fibroblast Basal Medium (ATCC PCS-201-030) and Fibroblast growth kit—low serum (ATCC PCS-201-041) for HGF cells, DMEM:F12 Medium (ATCC^®^ 30-2006™)—for SCC-4 cells were acquired from ATCC, and Dulbecco’s Modified Eagle Medium (DMEM) high glucose −4.5 g/L for HaCaT and A375 cells, together with the other reagents used, as: trypsin—EDTA solution, PBS (phosphate saline buffer), fetal bovine serum (FCS), Trypan blue, Alamar blue (resazurin sodium salt) and hydrocortisone were purchased from Sigma Aldrich (Darmstadt, Germany) and Thermo Fisher Scientific (Waltham, MA, USA).

### 3.4. Cell Culture

The HGF cells were grown in specific media—Fibroblast Basal Medium (ATCC PCS-201-030), supplemented with Fibroblast growth kit—low serum (ATCC PCS-201-041). SCC-4 cells growth required the use of DMEM:F12 Medium (Dulbecco’s Modified Eagle Medium) (ATCC^®^ 30-2006™) supplemented with 400 ng/mL hydrocortisone and fetal bovine serum (FCS) to a final concentration of 10%. HaCaT and A375 cells culture required specific media—Dulbecco’s Modified Eagle Medium (DMEM) high glucose 4.5 g/L, supplemented with 10% fetal bovine serum (FBS) and antibiotic mixture (100 U/mL penicillin and 100 μg/mL streptomycin). The cells were kept in a humidified incubator provided with 5% CO_2_ at 37 °C. The cells were numbered using a cell counting device—CountessTM II Automated Cell Counter (Thermo Fisher Scientific, Waltham, MA, USA), in the presence of Trypan blue.

### 3.5. Cell Viability Assessment

To assess the impact of the test compounds (single, binary, and ternary emulsions of CEO, OEO and BEO) on cell viability, the Alamar blue assay was conducted. The cells used in the study were healthy cells—immortalized human keratinocytes—HaCaT and human primary gingival fibroblasts—HGF, and tumor cells—human melanoma—A375 and human squamous tongue carcinoma cells—SCC-4. This assay was performed according to the following protocol: the cells were seeded in 96-wells plates (1 × 104 cells/well/ 200 μL) and let to acquire the appropriate confluence (24 h). Different concentrations of the test emulsions expressed as volume percentages % *v*/*v* were tested: 0.0031; 0.0625; 0.125; 0.250 and 0.625. The concentrations were obtained by dilution of the stock solutions 5 % into the fresh culture medium. The test compounds were maintained in contact with the cells for 24 h. After the 24 h period, it was added 20 μL of Alamar blue, followed by incubation for 3 h at 37 °C and measurement of the absorbance values at 570 and 600 nm by means of ×Mark™ Microplate Spectrophotometer (Biorad, Hercules, CA, USA). Cell viability assessment with Alamar Blue involves the evaluation of mitochondrial activity of living cells which reduce resazurin, a blue compound with maximum absorbance at 605 nm, to resorufin, a pink fluorescent compound that has the maximum absorbance at 573 nm [66].

Cell viability was calculated using the formula (1).

{[(ε_OX_)λ_2_ Aλ_1_ − (ε_OX_)λ_1_ Aλ_2_ of test agent dilution]/[(ε_OX_)λ_2_ A°λ_1_ − (ε_OX_)λ_1_ A°λ_2_ of untreated positive growth control]} × 100(1)

where ε_OX_ = molar extinction coefficient of Alamar blue oxidized form (BLUE); A = absorbance of test wells; A° = absorbance of positive growth control well (cells without tested compounds); λ_1_= 570 nm and λ_2_ = 600 nm

The coefficient of drug interaction (CDI) was used to analyze the interactions between the pure compounds while used as mixture. CDI was calculated as follows: CDI = AB/(A × B) where:

AB = absorbance for the mixture of the two active agents/absorbance value for the control

A and B = absorbance value for the single active agent/absorbance value for the control.

A CDI value <1, =1 or >1 indicates that the drug’s combination can either increase, produce an additive effect, or reduce, respectively, the effects produced by the same concentration of single drug. A CDI value less than 0.7 indicates that the drug’s combination of drugs significantly increases the effects, to a greater extent as compared to single drug testing or compared to the expected additive effect of the drug combination [66].

### 3.6. Cell Morphology

The effect of the first compounds on cells morphology was verified by taking pictures before addition of the first compounds and after the 24 h stimulation. The pictures were acquired by using the Olympus IX73 inverted microscope provided with DP74 camera photo and documented with the CellSens V1.15 software (Olympus, Tokyo, Japan).

### 3.7. The Skin Evaluation

The skin is an organ that responds very quickly to exposure to an aggressive external agent, such as solar radiation and some chemicals. In vivo evaluation of the irritative nature of the synthesized samples was performed by studying changes in erythema and hydration of the stratum corneum, and the barrier function of the skin was tested by transdermal water loss (TWL).

Eight healthy human subjects (3 men and 5 women, aged 22 to 37 years, mean: 28.5 years) were recruited in this study to evaluate the evidence obtained. The principles of the Helsinki Declaration and local jurisdiction were respected, and the volunteers read and signed informed consent. All determinations were performed with a multi-probe adapter system (Courage & Khazaka, Cologne, Germany) equipped with a Tewameter^®^ TM300 probe, a Mexameter^®^ MX18 probe, and a Corneometer^®^ CM825 probe. All 6 emulsions in concentration of 5% were used in skin evaluation.

### 3.8. Statistical Analysis

The statistical analysis was performed using GraphPad Prism v. 9.0 (GraphPad Software, San Diego, CA, USA). The Kolmogorov-Smirnov test was used for testing the distribution of variables. Variables with normal distribution were presented as mean value and standard deviation. The statistical differences were determined using two-way ANOVA analysis followed by Bonferroni post-test.

## 4. Conclusions

The in vitro approach implemented in the present study revealed the following results: (i) ECEO exerted a concentration-dependent cytotoxic effect, but it was unselective since the healthy and tumor cells were affected in such a manner; (ii) EBEO reduced cells’ viability percentage only at the highest concentration in skin cells (HaCaT and A375) whereas in the oral cells (HGF and SCC-4) triggered a dose-dependent decrease, (iii) EOEO had no toxic impact on skin cells, still the viability of oral cells was reduced after the highest dose applied, (iv) association of CEO and OEO emulsions determined a reduction of CEO toxicity and an increase in OEO cytotoxic effect both in healthy and tumor cells, (v) oral cells (HGF and SCC-4) showed an increased susceptibility to E(BEO/OEO) as compared to skin cells (HaCaT and A375), and (vi) the ternary emulsion E(BEO/CEO/OEO) reduced the antiproliferative effects compared to the effects of single EO on both healthy and cancer cell lines; therefore, it cannot act as anticancer agent but, can be safely applied on dermal or oral tissue thus being recommended as component of foods and beverages. The reduction of cells’ viability was accompanied by morphological changes that confirm the cytotoxic potential, such as: round and floating cells, reduced confluence, shrunken cells, and cell debris. The non-invasive measurements of physiological skin parameters indicate a safety profile of the test compounds.

Given the current trends regarding the use of complementary therapies based on EOs in the prevention and treatment of certain diseases, future studies of natural preparations on other cell lines are of future perspective.

## Figures and Tables

**Table 1:** Chemical composition (% of total) of EOs binary (BEO/OEO), (CEO/OEO) and ternary mixtures (BEO/CEO/OEO).

Nr.	Compounds	Type	Retention Time	LRI	% of Total		
					BEO/OEO	CEO/OEO	BEO/CEO/OEO
1.	α-pinene	MH	6.42	1013	**26.50**	-	20.15
2.	Camphene	MH	7.54	1057	0.58	-	0.43
3.	β-pinene	MH	8.68	1092	1.89	0.09	1.30
4.	Thujene	MO	9.04	1116	0.38	0.08	0.22
5.	β-myrcene	MH	10.21	1164	2.06	0.86	1.30
6.	4-carene	MH	10.75	1176	1.79	-	1.24
7.	D-limonene	MH	11.36	1189	**43.32**	**29.82**	**32.69**
8.	γ-terpinene	MH	12.65	1207	3.42		2.42
9.	*p*-cymol	MH	13.28	1212	2.43	0.04	1.70
10.	*p*-mentha-1,4(8)-diene	MH	13.72	1278	5.42	-	3.96
11.	α-terpinene	MH	13.90	1298	1.08	-	0.75
12.	1-hexanol, 4-methyl, acetate	MH	16.87	1489	0.38	-	0.23
13.	β -linalool	MO	20.90	1532	7.82	0.14	5.15
14.	α-caryophyllene	SH	22.53	1598	0.12	7.58	3.26
15.	α-terpineol acetate	MO	24.49	1643	0.70	1.90	0.49
16.	Benzyl alcohol		32.18	2071	-	26.75	8.76
17.	*p*-eugenol	MO	34.24	2192	**1.95**	**32.71**	**15.85**
**Total of Major Compounds**					99.84 *	99.97 *	99.90 *
Monoterpene hidrocarbonates (MH)					88.87	30.81	66.17
Monoterpene oxygenate (MO)					10.85	34.83	21.71
Sesquiterpene hidrocarbonates (SH)					0.12	7.58	3.26
Sesquiterpene oxygenate (SO)					-	-	-

* The difference up to 100% represents unidentified compounds (values not presented in the table).

**Table 2:** CDI values for binary and ternary mixtures.

Cell Type	CDI					
	E(BEO/OEO)		E(CEO/OEO)		E(BEO/CEO/OEO)	
	0.250%	0.625%	0.250%	0.625%	0.250%	0.625%
**HaCaT**	**0.4**	3.83	**0.74**	**0.87**	2.43	1.21
**A375**	**0.53**	1.68	1.09	**1.16**	1.1	2.57
**HGF**	**0.72**	**0.84**	-	-	-	-
**SCC4**	1.02	1.05	**0.82**	**0.93**	2.83	1.92

## Data Availability

The report of the analyzes performed can be found at the Research Center for Pharmaco-Toxicological Evaluations, “Victor Babeş” University of Medicine and Pharmacy, Timisoara, Romania.

## Abbreviations

EOs	essential oils
BEO	bergamot essential oil
CEO	clove essential oil
OEO	orange essential oil
EBEO	bergamot essential oil emulsion
ECEO	clove essential oil emulsion
EOEO	orange essential oil emulsion
E(BEO/OEO)	emulsion containing BEO and OEO
E(CEO/OEO)	emulsion containing CEO and OEO
E(BEO/CEO/OEO)	emulsion containing BEO, CEO and OEO
GC-MS	gas-chromatography coupled with mass-spectrometry
HGF	human primary gingival fibroblasts
SCC-4	human squamous cell carcinoma cell line
HaCaT	immortalized human keratinocytes
A375	human melanoma cells
